# A novel segmental autologous great saphenous vein grafting technique for size-mismatched traumatic common femoral artery reconstruction

**DOI:** 10.1016/j.jvscit.2026.102526

**Published:** 2026-09-28

**Authors:** Donglin Li, Huafeng Zheng, Sheng Wei, Lufeng Chen

**Affiliations:** Department of Vascular Surgery, The Second Affiliated Hospital of Fujian Medical University, Quanzhou, Fujian Province, China

**Keywords:** Femoral artery injury, Acute limb ischemia, Great saphenous vein, Reconstructive technique, Surgical management

## Abstract

Traumatic injury combined with common femoral artery occlusion often leads to acute lower-extremity ischemia. A 45-year-old man with left common femoral artery occlusion due to blunt trauma underwent emergency reconstruction. Given the severe caliber mismatch between the artery and great saphenous vein, we divided the vein into three segments and sutured them together to create an adequately sized conduit. Postoperative perfusion was excellent, and computed tomography angiography confirmed patency without stenosis. This simple, practical technique offers a useful alternative for femoral artery injury with size discrepancy, potentially reducing early graft failure and amputation in acute settings.

Traumatic femoral artery injury is rare but limb-threatening, accounting for 1% to 2% of all vascular traumas from penetrating or blunt mechanisms.^1^ It typically presents with acute ischemia, absent distal pulses, and sensorimotor deficits, yet diagnosis can be challenging in polytrauma patients with pelvic fractures, where the injury may be masked. Prompt computed tomography angiography (CTA) is critical for guiding emergent surgery and preventing irreversible damage.^2^ Traditional repair options include primary anastomosis, interposition grafting with synthetic conduits, or autologous vein grafts, but management is often complicated by extensive soft tissue damage, vessel wall contusion, and caliber mismatch between the native artery and available graft.^3^ The common femoral artery, in particular, is susceptible to blunt trauma and requires a conduit of adequate caliber.^4^ Autologous great saphenous vein (GSV) remains the preferred graft material for its infection resistance and patency, yet its smaller diameter frequently leads to turbulent flow, anastomotic stricture, and early thrombosis.^5^ Although spiral or panel grafts have been described, they demand prolonged operative time and specialized expertise, limiting their use in acute settings.

We report the case of a 45-year-old man with traumatic left common femoral artery occlusion from a crush injury. Emergency partial femoral artery resection and reconstruction were performed using a segmental GSV grafting technique: the vein was divided into three segments and sutured together to create a conduit of adequate diameter. Preoperative ultrasound localization and meticulous anastomosis contributed to excellent immediate perfusion, and follow-up CTA confirmed graft patency. This case illustrates a practical and effective strategy for managing size-mismatched femoral artery injuries, offering a useful reference for similar complex reconstructions in the trauma setting. The patient provided informed consent for treatment and publication of this case report and accompanying images. No protected health information was disclosed, and no institutional review board approval was required.

## Case report

A 45-year-old man with lower-limb trauma and a pelvic fracture presented with acute left lower-limb ischemia. The time of the injury was 2 hours. The patient exhibited significant clinical signs of acute arterial insufficiency in the left lower limb, including reduced sensory perception, and decreased motor strength. Physical examination before surgery revealed absent pulses in the left femoral and popliteal arteries. CTA revealed a hematoma in the left inguinal region and nonvisualization of the left common femoral artery (Fig 1, *A* and *B*). Based on these findings, we considered that the left common iliac artery had ruptured.

**Fig 1:**
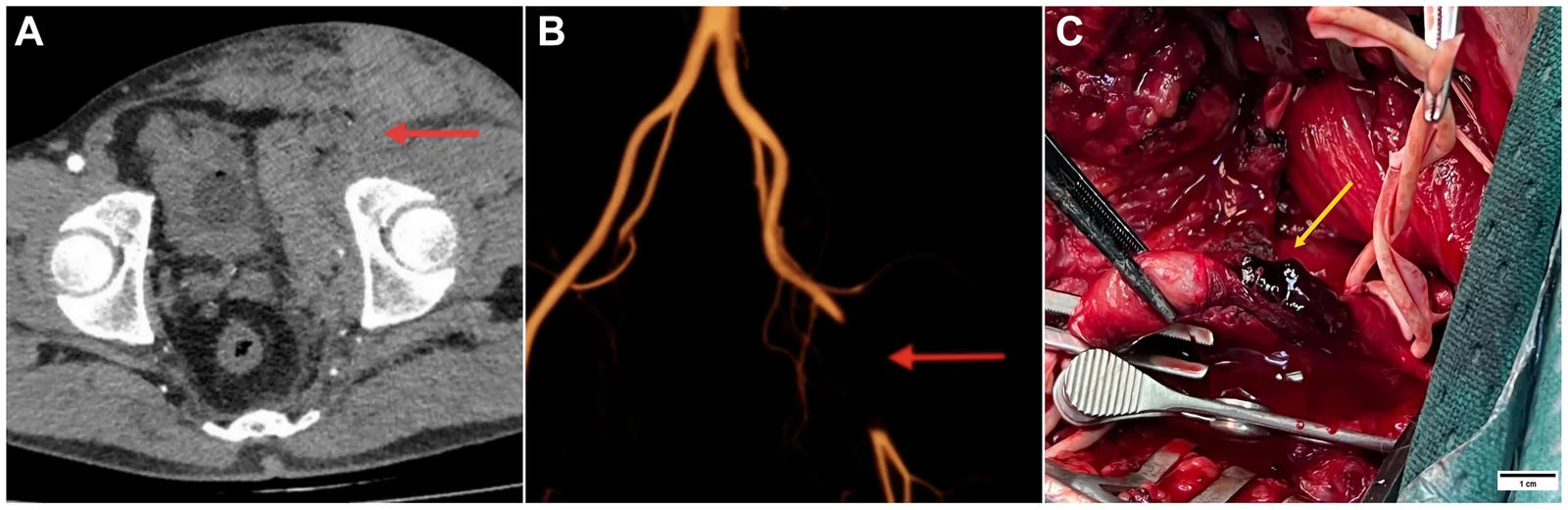
Preoperation **(A-C)**. **A,** The computed tomography angiography (CTA) scan showed that the left common femoral artery did not show up (*red arrow*). **B,** CT three-dimensional (3D) reconstruction showed occlusion of the left common femoral artery (*red arrow*). **C,** Intraoperative view showing a rupture of the left common femoral artery (*yellow arrow*). Scale bars in **(C)** indicate [X] cm (calibrated using ImageJ software).

The patient had developed acute ischemia in the left lower limb. After careful consideration, it was necessary to perform an emergency surgical exploration of the left femoral artery. The patient underwent an emergency surgical procedure under general anesthesia. After standard surgical exposure of the left femoral artery, significant hematoma was evacuated, revealing the injured segment of the common femoral artery. The arterial wall at the injury site was edematous and fractured, with no palpable pulse noted locally or distally (Fig 1, *C*).

As the patient and his family requested the use of autologous veins for the transplantation, we decided to use autologous saphenous vein grafts for the repair of the left common femoral artery. Before femoral artery clamping, systemic heparin is administered and the activated clotting time is measured to ensure adequate anticoagulation. The activated clotting time was monitored and maintained at >250 seconds throughout the reconstruction. Under ultrasound guidance, the main trunk of the left GSV was harvested (Fig 2, *A*). Given the relatively large caliber of the patient’s native femoral artery compared with the harvested vein, the saphenous vein segment was divided into three sections, which were then sutured together to create a single conduit with a diameter approximating that of the native artery (Fig 2, *B* and *C*). The three longitudinal suture lines were created using a simple running 7-0 Prolene continuous suture technique, with careful attention to full-thickness incorporation and slight eversion of the edges to promote intima-to-intima apposition. The injured segment of the femoral artery was resected to healthy margins. The reconstructed autologous saphenous vein graft was then anastomosed end-to-end to both the proximal and distal ends of the native femoral artery. Upon release of the arterial clamps, there was no evidence of leakage at the repair site, and a strong pulse was palpable in the distal femoral artery (Fig 3, *A*). Hemostasis was achieved, and the wound was closed in layers. The procedure was completed smoothly with minimal blood loss, and the patient was returned to the ward in stable condition.

**Fig 2:**
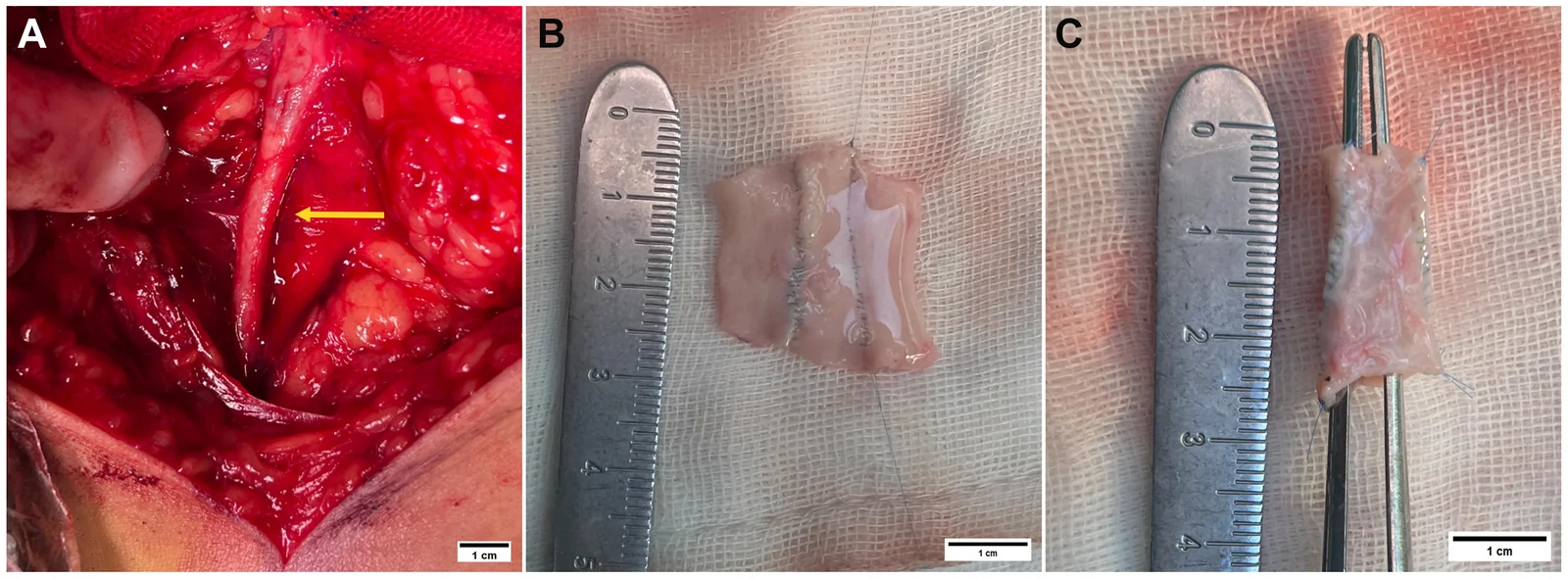
During operation **(A-C)**. **A,** The great saphenous vein was noted to be of small caliber (*yellow arrow*). **B,** The long sides of these three segments were sutured side by side with 7-0 Prolene continuous sutures. **C,** The manually created graft was fashioned to a 10 mm diameter (1 mm larger than the left common femoral artery). Scale bars in **(B)** and **(C)** indicate [X] cm (calibrated using ImageJ software).

**Fig 3:**
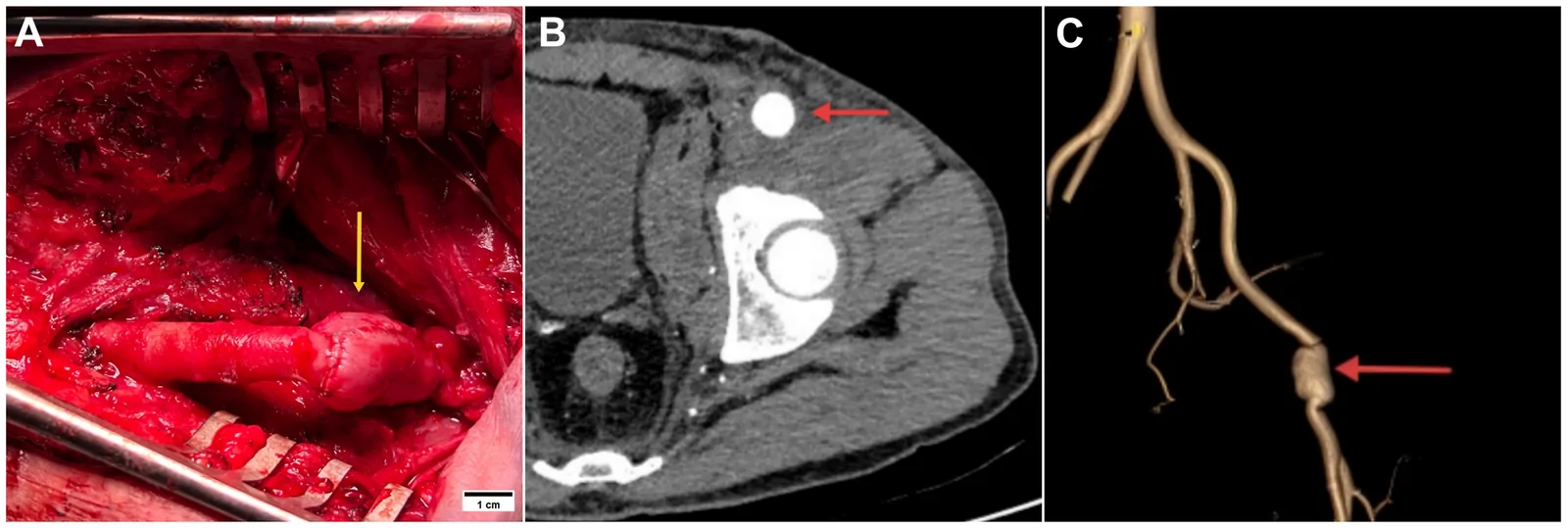
Postoperation **(A-C)**. **A,** Intraoperative view showing a patent reconstruction of the left common femoral artery with good caliber and flow (*yellow arrow*). **B** and **C,** Postoperative computed tomography angiography demonstrating the blood flow of the left common femoral artery was unobstructed (*red arrow*). Scale bars in **(A)** indicate [X] cm (calibrated using ImageJ software).

Postoperatively, the ischemic symptoms in the patient's left lower limb disappeared. The left femoral and popliteal pulses, which were absent before surgery, were now palpable and strong. The surgical drain was removed on the third postoperative day. A follow-up CTA performed on the fourth postoperative day confirmed patency of the left femoral artery with good blood flow and no evidence of contrast extravasation (Fig 3, *B* and *C*). The patient received rivaroxaban 20 mg once daily starting on postoperative day 1, continued through discharge and planned for 2 weeks. This regimen was chosen based on the favorable safety profile of direct oral anticoagulants in peripheral arterial procedures and the reduced bleeding risk compared with warfarin. The patient was also started on aspirin 100 mg daily as antiplatelet therapy 2 weeks later, and the treatment was planned for 6 months.

## Discussion

We present a case of blunt traumatic occlusion of the left common femoral artery in a 45-year-old man, successfully managed by segmental resection and reconstruction using a modified autologous GSV grafting technique tailored to a pronounced vessel diameter mismatch. This approach diverges from conventional interposition grafting. Bovine pericardium represents a potential alternative biologic conduit that could offer excellent size match and require only a single suture line, potentially reducing construction time. Although we favored autologous vein in this case to honor the patient’s preference and avoid xenograft-related costs, bovine pericardium may be a valuable option when autologous vein is unavailable or insufficient, particularly in the acute trauma setting. In a retrospective series of 77 traumatic lower-extremity arterial injuries, autologous GSV bypass was a standard option, yet one case developed 30% distal anastomotic stenosis.^6^ Our technique—splitting the vein into three longitudinal segments and resuturing them into a larger-caliber conduit—may reduce such size-related complications, there had also been reports using this method to reconstruct the popliteal venous aneurysm.^7^ Alternative strategies, such as spiral saphenous vein grafting, have been proposed for diameter-mismatched arterial reconstruction, with evidence indicating that patent grafts preserve vascular identity plasticity.^8^ Likewise, a bifurcated GSV segment used as a vascular loop in lower-limb reconstruction illustrates the vein’s adaptability to specific anatomical demands.^9^ The excellent short-term patency observed in our patient, confirmed by postoperative CTA, aligns with favorable long-term outcomes reported for GSV grafts in diverse arterial settings, including a 20-year patent graft after right coronary artery aneurysm repair and successful anatomical coronary reconstruction in children.^10^^,^^11^

We acknowledge that in a short-segment, noncontaminated common femoral artery injury, a ringed polytetrafluoroethylene graft would be a reasonable alternative with excellent expected patency. The decision to use an autologous vein graft in this case was influenced by the patient’s and family’s preference to avoid prosthetic material, the patient’s young age, and the potential for long-term infection resistance with autogenous conduit. However, we recognize that this technique should be considered one useful option among several rather than a universally preferred approach, and the choice of conduit should be individualized based on patient factors, injury characteristics, and surgeon expertise. In this case, the graft was sized to approximately 10 mm in diameter using a sterile graduated ruler, which was 1 mm larger than the native common femoral artery (approximately 9 mm). In retrospect, a more precise caliber match might have been achieved using a 24F Foley catheter or chest tube as a sizing mandrel, as suggested by the reviewer. This technique could facilitate more accurate diameter matching and potentially reduce the risk of turbulent flow or graft redundancy. Although two vein segments might have been sufficient, we elected to use three segments to ensure a comfortable margin and avoid the risk of an undersized conduit.

This case also emphasizes pivotal diagnostic and technical principles. Pelvic fracture, a marker of high-energy trauma, should prompt a high suspicion for occult vascular injury—a concern reinforced by studies linking pelvic fractures to mortality from missed arterial lesions.^12^ Preoperative color Doppler ultrasound enabled precise localization of both the injured arterial segment and the suitable GSV donor site, minimizing dissection and operative time. Such meticulous planning is cornerstone in complex vascular repairs, as demonstrated in transplant renal artery aneurysm and superior mesenteric artery aneurysm reconstruction.^13^^,^^14^ Although single-segment GSV interposition is common, our segmental reconstruction directly confronts the challenge of a markedly larger native artery, wherein a standard vein graft may predispose to turbulent flow and thrombosis. This innovative adaptation—analogous to using a Y-shaped graft for dual-vessel supply in lower-extremity salvage—offers a valuable strategy for managing caliber mismatch in traumatic arterial injuries, potentially enhancing early patency and limb preservation.^15^

Several salient lessons emerge, particularly concerning diagnostic pitfalls and the pathophysiology of blunt arterial injury. A major challenge is that a life-threatening vascular lesion may be overshadowed by concomitant orthopedic trauma, as exemplified by the pelvic fracture in this patient. Clinicians must remain vigilant for vascular compromise even when obvious skeletal injuries dominate; the classic signs of acute limb ischemia—pain, pallor, pulselessness, paresthesia, and poikilothermia—can be subtle or falsely attributed to the fracture itself.^1^^,^^16^ The mechanism in our case, high-energy blunt force to the groin, typifies “handlebar” or “motor-scooter” syndrome, which can produce intimal flaps, thrombosis, or even complete transection of the common femoral artery without an associated fracture.^17^^,^^18^ This matches our intraoperative findings of a thrombosed, contused segment. The pathological spectrum ranges from intimal disruption and intramural hematoma to total occlusion, mandating complete resection of the damaged portion to avert subsequent thrombosis or pseudoaneurysm formation.^19^ Delayed recognition, as documented in missed femoral injuries, can precipitate catastrophic sequelae—compartment syndrome, rhabdomyolysis, and limb loss—underscoring the imperative for a rigorous vascular examination and liberal CTA use in any patient sustaining high-energy lower-extremity trauma.^20^^,^^21^ The choice of open surgical repair over endovascular intervention was also critical, whereas stenting is increasingly applied to superficial femoral artery injuries, its role in the common femoral artery—particularly with active thrombosis and a contused wall—remains limited, and open reconstruction is generally preferred for definitive management and long-term durability.^22^

## Conclusions

This case underscores the importance of rapid CTA-based diagnosis and flexible intraoperative strategy in complex vascular injuries. To overcome the significant caliber discrepancy, we used a customized autologous vein graft constructed by splitting and resuturing the GSV into three segments—a technique that circumvented the limitations of standard single-segment conduits. Although effective in this instance, this approach requires further validation; the present report is limited by its single-case nature and cannot establish the generalizability or long-term durability of this method. Future cohort studies with extended surveillance are warranted, and the optimal perioperative antithrombotic protocol remains a critical unanswered question.

## Funding

None.

## Disclosures

None.
